# Highlights of the *e*cancer/SAC First International Prostate Cancer Symposium, 11–12 March 2016, Buenos Aires, Argentina

**DOI:** 10.3332/ecancer.2016.640

**Published:** 2016-05-05

**Authors:** Marcelo Blanco Villalba, Marina Bramajo, Mario Bruno

**Affiliations:** Sociedad Argentina de Cancerología (SAC), Av Santa Fe 1171, Buenos Aires, C1059ABF, Argentina

**Keywords:** prostate, urology, imaging, radiotherapy

## Abstract

The *e*cancer/SAC First International Prostate Cancer Symposium, held in Buenos Aires, included national, regional, and international experts in the field of prostate cancer.

More than 200 professionals from a variety of areas (clinical urologists, pathologists, oncologists, biologists, imaging specialists, radiation therapists, and generalist doctors, among others) attended, and they proposed multidisciplinary management of prostate pathology from the start in concordance with the ideas set forth by the organising committee.

A radiotherapy workshop was also held during the symposium, in which new techniques and their possible uses were specifically discussed. In addition to the local doctors, Dr Lilian Faroni (COI Group, Rio de Janeiro, Brazil), Dr Leonardo Carmona (Chilean Head and Neck Institute, Chile), and Dr Anthony Addesa (Jupiter Medical Centre, Florida, USA) also participated in this symposium.

## Introduction

The main objective of *e*cancer/SAC’s First International Prostate Cancer Symposium was to provide an approach for dealing with the issue of prostate cancer in South America as well as to review the latest scientific knowledge in order to propose multidisciplinary treatment which would allow results and resources to be optimised.

The main issues that were highlighted include: 1) epidemiology and the prevention of prostate cancer; 2) surgical treatment and radiotherapy; 3) further consideration for the assessment of both pathological anatomy as well as imaging; 4) updating systemic treatment.

## Epidemiology and the prevention of prostate cancer

UICC past president Dr Eduardo Cazap of Buenos Aires, Argentina opened the presentation. He started off by highlighting the difficulty one faced while collecting data in the region, and later on he said that this situation will improve because of the joint work being carried out by the regional and National Cancer Institutes for obtaining the data. With the current data, cancer mortality is greater in Latin America than in the US and Europe, and Dr Cazap suggested using the mortality/incidence rate to monitor the results of health policies [1]:

**Table:** 

	Latin America	US	Europe
Mortality/Incidence:	0.59	0.43	0.35
Mortality-Argentina*:	3.937/year 2010. Second leading cause in men (after lung cancer).		

From regional presentations it emerged that the fundamental question continues to be, ‘Who should be treated and when?’ Recommendations differ: in Chile, screening begins at age 45, while in Argentina it begins at age 50. In Bolivia, its incidence is low (2.2%). Given these different realities and data still being in progress, it is difficult to develop a common regional strategy.

## Surgical treatment and radiotherapy

For localised tumours, Dr Wenceslao Villamil presented the possibilities of robotic surgery based on the work of his team at the Hospital Italiano de Buenos Aires, while Dr Carlos Ameri of the Sociedad Argentina de Urología (SAU) emphasised the multidisciplinary management of these patients and the need to individualise them in order to choose a course of treatment.

On the other hand, Dr Leonardo Carmona of Chile commented on new external radiotherapy technologies for prostate cancer, Dr Luisa Rafailovicci of the Vidt Medical Centre in Buenos Aires referred to the association of radiotherapy and hormonal therapy in patients with locally advanced intermediate-high risk.

Dr Pablo Castro Rock of the Institute of Radiotherapy-Marie Curie Foundation in Córdoba, Argentina mentioned the possibility of using brachytherapy in select patients. Finally, Dr Silvia Zunino, director of the same institute, raised the controversial issue of irradiation of the ganglia in the treatment of locoregional prostate cancer, concluding that it should not be standard practice and should be reserved for the group of patients with the worst prognosis.

## Renewed consideration for the assessment of both pathological anatomy and imaging

Four sessions were dedicated to this topic. Prof Alberto Lazarowski of the University of Buenos Aires (UBA) spoke about molecular biology and pharmacogenetics in prostate cancer, presenting the range of possibilities which is opened up by liquid biopsy both to assess the situation as well as to evaluate treatment response, which would lead to an optimisation of results.

Dr Laura Jufe and Dr Claudio Lewin of the Sociedad Argentina de Patología (SAP) mentioned the changes that were recently made to the Gleason score, which they took to the World Health Organisation (WHO) this year to change the classification. It will be denominated by group: the traditional Gleason 3 + 4 (score 7) according to histopathological characteristics will convey the same information as Group 2 or 3, as the prognosis and therapeutic treatments differ, such as active surveillance among others.

Dr. Martin Eleta of the IMAXE Imaging Centre in Buenos Aires talked about new techniques for early identification of metastasis, presenting a casuistry of more than 600 patients where the use of choline PET improved staging in terms of sensitivity and specificity versus CT and scintigram, which by identifying early metastasis changed therapeutic treatment by 20% [2].

## Update on systemic treatment

During this session, Dr Gustavo Jankilevich, head of the oncology service at the Carlos Durand hospital in Buenos Aires, spoke about opportunities for the use of hormonal therapy both in terms of when to begin as well as on the controversial topic involving duration of hormone blocking. He stressed the need to make an appropriate selection of patients to treat—using chemotherapy in a biochemical relapse situation accompanied by high tumour volume while conversely evaluating a combination with hormone therapy if it is accompanied by low tumour volume.

Dr Juan Pablo Sade of the Alexander Fleming Institute in Buenos Aires mentioned that aside from talking about new therapeutic targets, we should refer to known drugs that have optimised their mechanisms of action allowing ‘a resurgence of the role of chemotherapy in prostate cancer’.

Finally, Dr Ricardo Kirchuck (representing the National Cancer Institute of Argentina, INC) and Dr Maria Bastianello (Centre for Medical Education and Clinical Research in Buenos Aires, CEMIC) outlined the management of bone metastasis with the use of radiopharmaceuticals including radium 223 [3].

## Access to health services

In the last session, the subject of access to health services was considered. According to estimates, in South America by 2030 it is expected that the percentage of deaths attributable to non-communicable diseases (NCD) will rise to 81% of total deaths [4]. Here we see that the need for development of a regional prevention policy would be fundamental (with each country’s National Cancer Institute being the natural organisation for this task). On the other hand, we must also introduce into professional training curricula the concept that disparities do have consequences, i.e. as we note that the probability of a newborn dying in the province of Formosa (Argentina) is 2.5 times greater than in Buenos Aires [5].

If we also look at expenditure on oncology treatments (% of the GNP) [6]:

– US: 1.02%
– Japan: 0.60%
– United Kingdom: 0.51%
– Latin America: 0.12%. *(Uruguay: 0.29%, Argentina: 0.16%, Venezuela: 0.06%)*

We find that disparities are ‘the real world’ in our subcontinent.

In our region, we have had the most of the advances both in terms of assessment as well as treatment, but unevenly. One of the noteworthy feature in South America is that there are large areas of countries with heterogeneous population densities, leading to unequal ‘access’ between countries and also within a single country.

This is why optimisation of resources is fundamental (the region’s installed capacity is sizable, but is generally concentrated in large urban centres), with personalised therapy through multidisciplinary management being one of the most useful tools to achieve what Dr Margaret Chan expressed at the 65th World Health Assembly (United Nations, May 2012): ‘Universal health coverage is the single most powerful concept that public health has to offer.’

## Conclusion

From the current data we can observe that, with respect to prostate cancer, our current difficulties continue in a similar way to previous years in terms of prevention and treatment. In order for the recommendations to become valid, it is fundamental that the actions of scientific societies are carried out. Regarding treatment, a lot of emphasis was made on the importance of multidisciplinary treatments and personalized medicine for patients. Finally the issue of regional disparities will be the axis to optimize resources to obtain better results for our patients.
